# Endovascular Treatment of a Multi-visceral Aortic Conduit Blowout With Parallel Stent Grafts and Coils

**DOI:** 10.7759/cureus.53707

**Published:** 2024-02-06

**Authors:** Dimitrios Moris, Mitchell W Cox, Zachary Williams

**Affiliations:** 1 Department of Surgery, Duke University Medical Center, Durham, USA; 2 Department of Vascular Surgery, University of Texas Medical Branch at Galveston, Galveston, USA; 3 Department of Vascular Surgery, Duke University Medical Center, Durham, USA

**Keywords:** endovascular, graft, stent, coil, multivisceral transplant

## Abstract

Multi-visceral transplantation (MVT) is a complex surgical procedure involving the transplantation of multiple abdominal organs as a single unit, typically used as bailout treatment of patients with devastating abdominal pathologies. Due to the complexity of the procedure, major and even life-threatening complications can happen. Vascular complications, including anastomotic breakdowns or pseudoaneurysms due to infections, can be universally lethal. Open surgical repair is often not an option due to the hostile operative field. We report a case of endovascular salvage of multi-visceral aortic conduit blowout utilizing parallel stent grafts and coils without sacrifice of the transplanted viscera. This combination can successfully control bleeding and maintain graft perfusion in this rare but devastating complication.

## Introduction

Multi-visceral transplantation (MVT), also known as composite visceral transplantation or multiorgan transplantation, is a complex surgical procedure involving the implantation of multiple abdominal organs as a single unit, which typically includes the liver, pancreas, stomach, and small intestine and sometimes other organs such as the colon [1]. It is usually performed in cases where patients have extensive abdominal organ failure or diseases affecting multiple organs (short gut syndrome, intestinal failure, etc.).

Due to the complexity of MVT, complications are relatively common, mainly attributed to vascular complications, infections, and immunologic complications. Anastomotic breakdown or pseudoaneurysm is a universally lethal complication attributed to infections of the graft; thus, they require urgent repair. The incidence of arterial bleeding can be as high as 5% of cases and can pose a great challenge to surgeons involved due to the complexity of vascular reconstructions used for MVT and the presence of a hostile surgical field [2-4]. Thus, open repair is difficult, and its outcomes might be poor with many patients dying of graft loss or re-bleeding [2,3]. Anastomotic pseudoaneurysms are particularly challenging as rupture is typically fatal and attempts at repair often lead to graft loss or thrombosis and patient death. Endovascular techniques are appealing because they allow the exclusion of the pseudoaneurysm and control of the bleeding with minimal interruption in transplant organ perfusion. This technique also avoids an extremely challenging dissection in the setting of uncontrolled aortic bleeding. This report describes a case of endovascular salvage of MVT aortic conduit blowout utilizing parallel stent grafts and coils without compromising the transplanted viscera.

## Technical report

**Figure 1:**
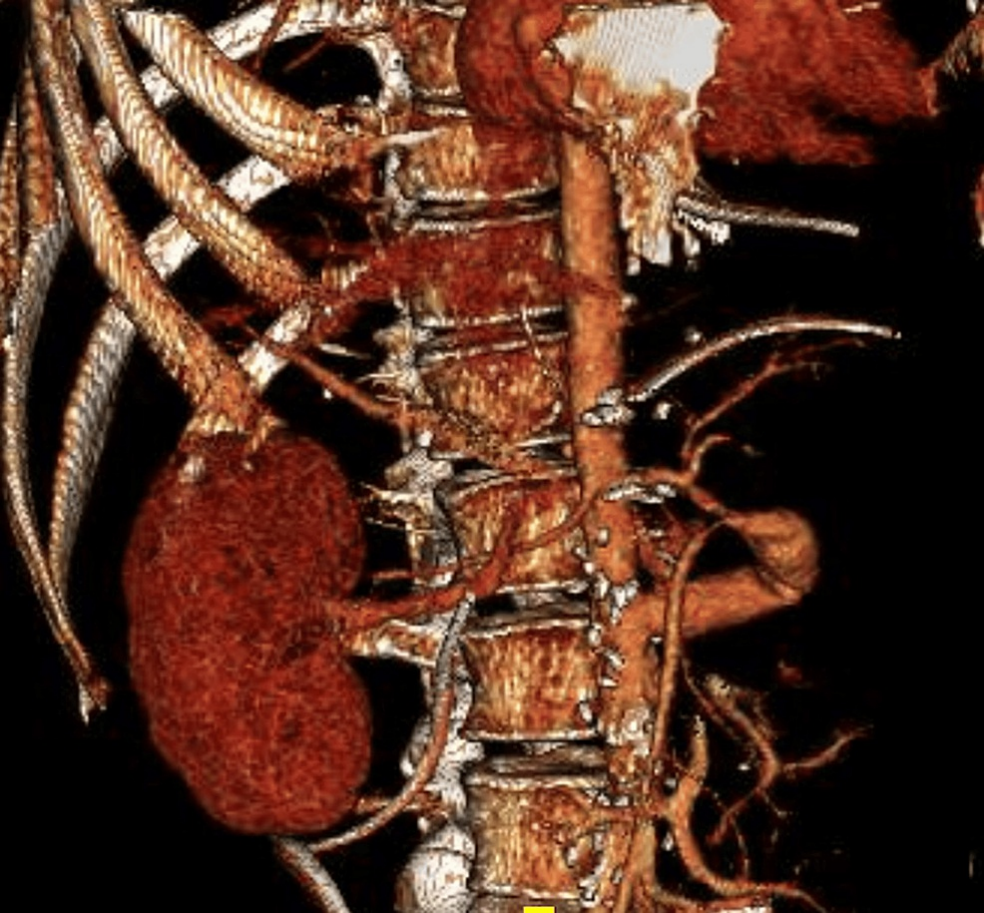
3D volume-rendered images demonstrating altered arterial anatomy with the visceral vessels being supplied via an infrarenal aortic conduit

A 45-year-old male with a remote history of Hodgkin lymphoma was treated with splenectomy and radiation therapy. He suffered multiple complications including cirrhosis, radiation enteritis requiring parenteral nutrition, and duodenal obstruction. He ultimately underwent an MVT of the liver, pancreas, stomach, small intestine, and right colon. The arterial reconstruction of donor viscera was accomplished via an end-to-side anastomosis of the infrarenal native aorta and donor thoracic aorta (Figure 1).

**Figure 2:**
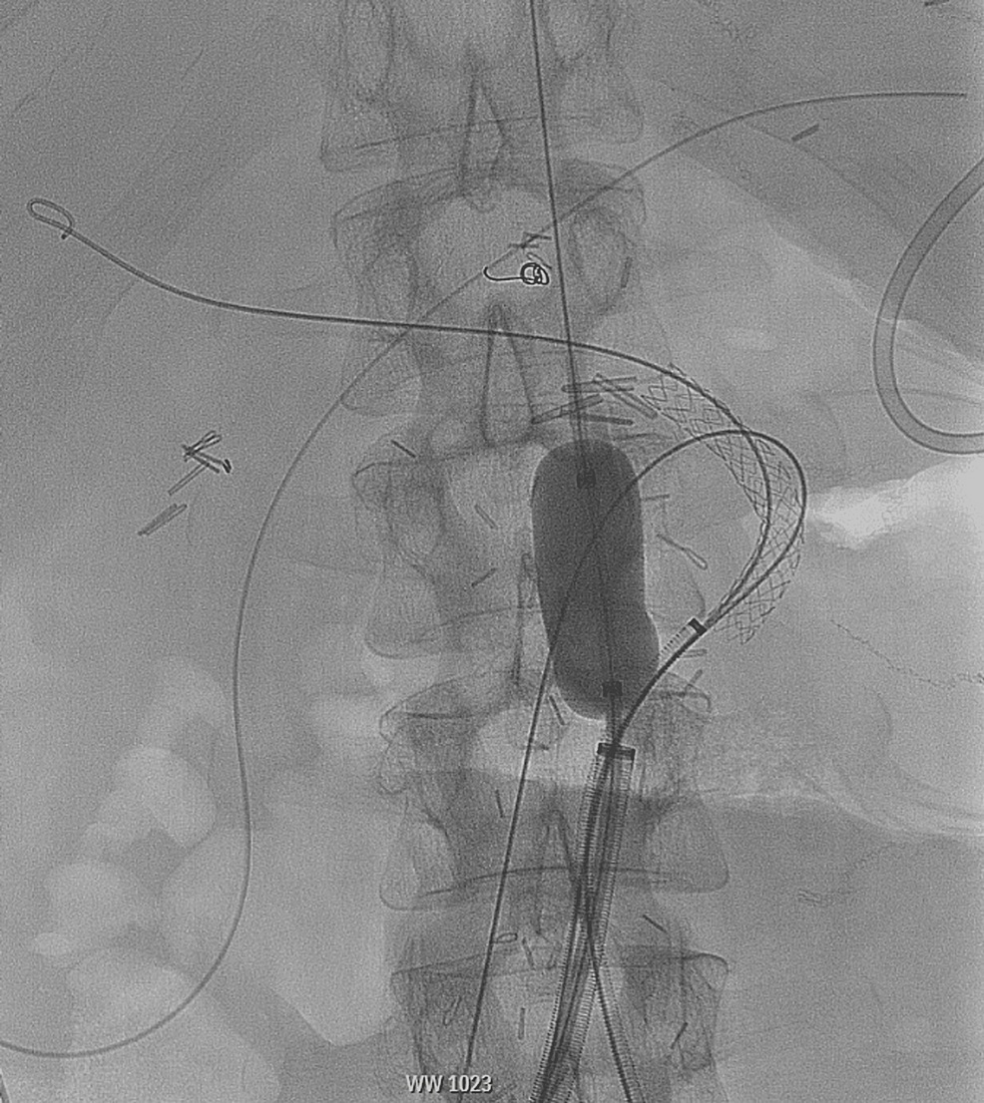
Intraoperative angiogram showing Coda balloon in the native aorta assisted with hemostasis during stent graft placement

Postoperatively, the patient developed severe necrotizing pancreatitis requiring multiple takebacks and debridements. During one of the re-explorations, significant bleeding was noted from the aortic conduit. Vascular surgery was intraoperatively consulted. Upon exploration, it appeared that the donor aorta was bleeding due to infection and a durable open surgical repair would be impossible due to the open abdomen, pancreatitis, infection, and prior transplant. An endovascular approach was pursued, and the patient was transferred to the hybrid suite. Bilateral femoral arterial access was obtained, and an aortogram was performed to better delineate the anatomy. An 18-Fr DrySeal (W.L. Gore & Associates, Inc., Flagstaff, AZ) was placed in the native aorta, and it was doubly accessed with two 7-Fr ANL sheaths (Cook Inc., Bloomington, IN). The aortic conduit was accessed, and a wire was advanced into the superior mesenteric artery. As the celiac artery was selected, massive bleeding was noted from the open abdominal incision, and it was clear that the donor aorta had ruptured, creating an aorto-atmospheric fistula. Temporary hemostasis was obtained with a Coda balloon in the native aorta and pledgeted sutures in the area of rupture (Figure 2).

**Figure 3:**
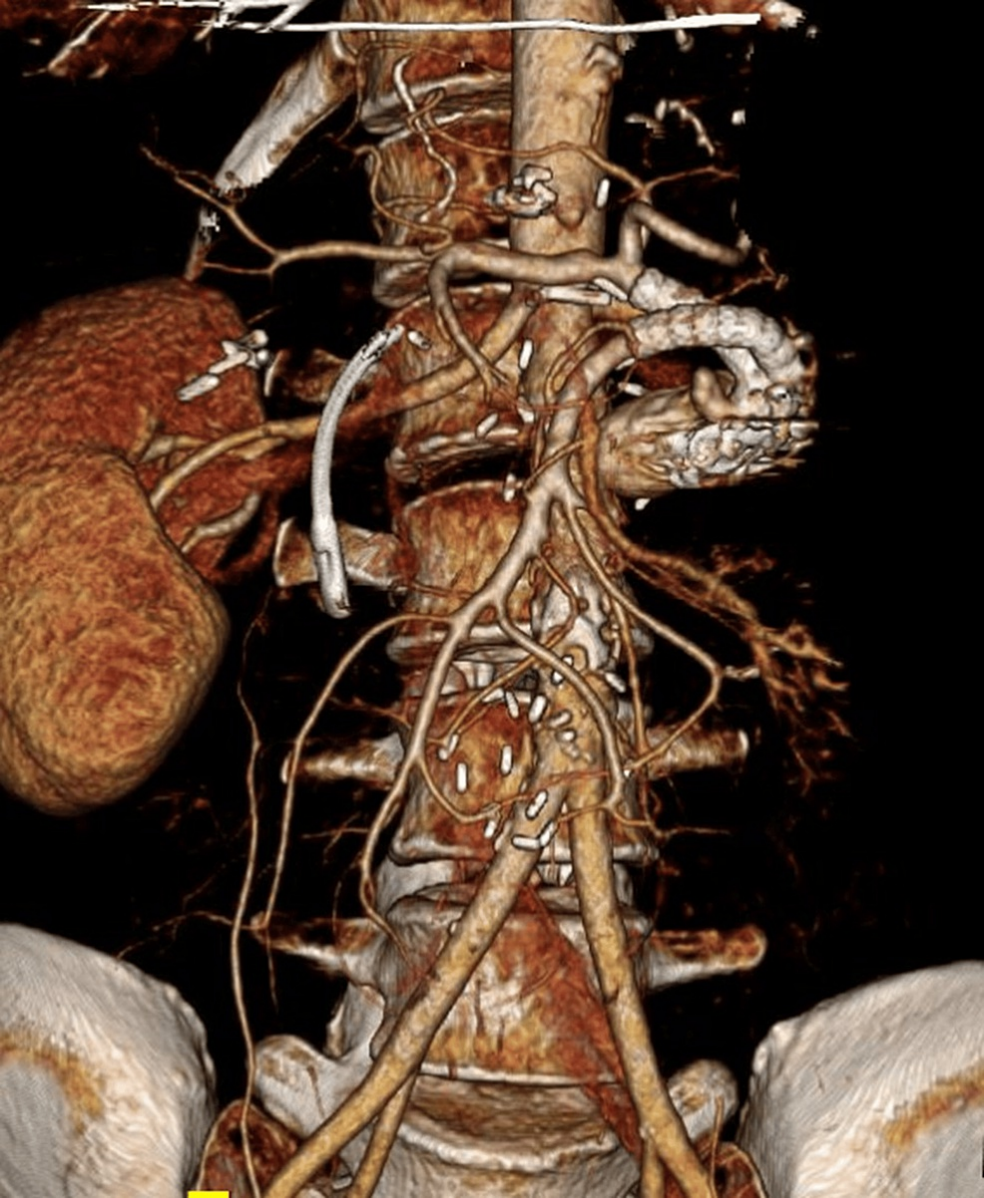
Postoperative imaging after parallel stent grafting and coil embolization of the aortic conduit

Leaving the Coda balloon inflated, the celiac artery was then re-selected. Two parallel covered stents (GORE® VIABAHN® VBX), one in the celiac artery and the other in the superior mesenteric artery, were deployed. Large coils were then deployed around these stents to try and seal any gutter leak. This achieved hemostasis (Figure 3).

Completion angiogram demonstrated excellent flow to the superior mesenteric artery and celiac artery with no contrast extravasation. The patient remained critically ill with incremental improvement and without any further bleeding episodes for the next month. At that time, surveillance cross-sectional imaging revealed a new pseudoaneurysm originating from the conduit.

**Figure 4:**
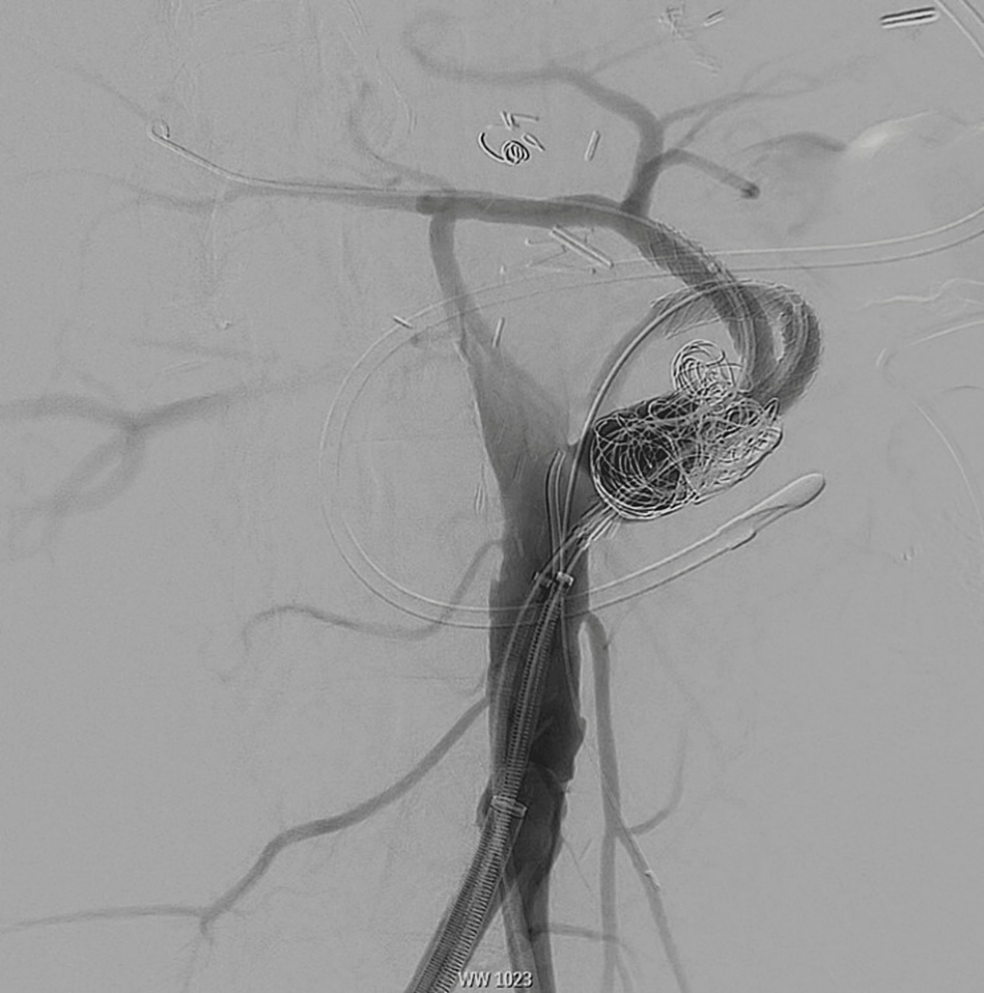
Intraoperative angiogram showing stent extension and additional coil placement up to the native aorta

The patient was taken back to the hybrid suite. Bilateral femoral arterial access was obtained, the celiac artery and superior mesenteric artery stents were cannulated, and the 7-Fr ANL sheaths were left in place. Several large coils were placed in the aortic conduit around the sheaths. Additional covered stents (GORE® VIABAHN® VBX) were deployed, extending the previously placed celiac artery and superior mesenteric artery stents up to the native aorta. Completion angiogram revealed good flow in the superior mesenteric artery and celiac artery without any filling of the pseudoaneurysm (Figure 4).

Multiple intraoperative cultures and blood cultures grew *Candida glabrata*. After an 18-week hospitalization, the patient was ultimately discharged home remaining on suppressive isavuconazonium. Months later, he was readmitted with pneumonia and failure to thrive, ultimately requiring a tracheostomy. He ultimately elected to transition to comfort care and passed away 10 months after his initial repair.

## Discussion

Multi-visceral transplantation is a complex surgical procedure involving the transplantation of multiple abdominal organs as a single unit, typically used as bailout treatment of patients with devastating abdominal pathologies. Starzl et al. performed the first MVT in 1987, and since then, there has been significant evolution of the procedure and its outcomes [5]. The arterial reconstructions performed for MVT use a donor thoracic aortic conduit sewn end-to-side to the native recipient infrarenal aorta [4]. Arterial bleeding due to conduit ruptures is extremely uncommon but fatal complications. When occurring late, as in our case, they may be associated with bacterial or fungal infection [2,3,6,7]. Emergent open surgery for damage control and repair of these life-threatening complications is challenging due to the hostility of the abdomen, often attributed to infection, desmoplastic reaction, or adhesive disease. Hybrid or endovascular techniques using wires, balloons or stents, coils, hydrogels, and thrombin have been proposed [8,9], but cases still require multiple takebacks due to re-bleeding events [2].

Pure endovascular access and management do not require exposure of the abdomen, but the complexity of the vascular anatomy in these cases is challenging. The morphology and etiology of the arterial blowout can limit the use of embolization, especially in the setting of large vessels such as the aortic conduit that was involved in our case. Managing a stump rupture from a previous MVT with aortic endograft has been previously reported [2]. Commercially available fenestrated aortic endografts are inadequate to adjust to the post-MVT anatomy. However, the use of physician-modified fenestrated endografts for the treatment of visceral transplant pseudoaneurysms has been reported [10]. Aortic endograft placement in conjunction with chimney or snorkel grafts for visceral perfusion has also been described and remains an option in this challenging situation [11,12].

## Conclusions

Multi-visceral transplantation is a complex surgical procedure involving the transplantation of multiple abdominal organs as a single unit, typically used as bailout treatment of patients with devastating abdominal pathologies. These are challenging cases requiring complex arterial reconstructions. Anastomotic blowout or pseudoaneurysm formation attributed to infection is a universally lethal complication. In these patients, open surgical repair is often not an option due to the hostile operative field. The combination of endovascular parallel covered stent grafting and coil embolization can successfully control bleeding and maintain graft perfusion in this rare but devastating complication.
